# Are weak dispersers more vulnerable than strong dispersers to land use intensification?

**DOI:** 10.1098/rspb.2022.0909

**Published:** 2023-01-11

**Authors:** Amanda E. Martin, Jessica K. Lockhart, Lenore Fahrig

**Affiliations:** ^1^ Environment and Climate Change Canada, National Wildlife Research Centre and Department of Biology, Carleton University, Ottawa, Ontario, Canada K1S 5B6; ^2^ Geomatics and Landscape Ecology Laboratory, Carleton University, Ottawa, Ontario, Canada K1S 5B6

**Keywords:** anthropogenic land use, habitat fragmentation, habitat loss, migration, mobility, vagility

## Abstract

Ecologists often state that weak dispersers are particularly at risk from land use intensification, and that they therefore should be prioritized for conservation. We reviewed the empirical evidence, to evaluate whether this idea should be used as a general rule in conservation. While 89% of authors predicted that weak dispersers are more vulnerable to land use intensification (80 out of 90 papers), only 56% of reported tests (235 out of 422) were consistent with this prediction. Thirty per cent of tests (128 out of 422) were consistent with the opposite prediction, that strong dispersers are more vulnerable to intensification, and 60% of articles (45 out of 75) had at least one test where strong dispersers were most vulnerable. The likelihood of finding that weak dispersers are more vulnerable to intensification than strong dispersers varied with latitude, taxonomic group and type of land use intensification. Notably, the odds of finding that weak dispersers are more vulnerable to intensification than strong dispersers was higher if the study was nearer to the equator. Taken together, our results show that the prediction that weak dispersers are more vulnerable than strong dispersers to intensification is not sufficiently supported to justify using weak dispersal as a general indicator of species risk in human-modified landscapes.

## 1. Introduction

Species with weak dispersal are often thought to be particularly at risk from land use intensification (hereafter ‘intensification’), such as urbanization, road expansion and clearing for agriculture [1–3]. Thus, weak dispersers are predicted to show greater population declines, extinction probabilities and range contractions than strong dispersers in the face of intensification. Weak dispersers that rarely move among remnant habitat patches should have small, isolated populations that are at high risk from processes such as inbreeding depression and demographic stochasticity [4–6]. By contrast, strong dispersers should be more able to move among habitat patches, allowing rescue of small populations and recolonization after local extinctions [7,8]. In addition, if intensification degrades the quality of remaining habitat, individuals may need to access more habitat to compensate for reduced habitat quality [9–11]. Strong dispersers may be better able to compensate for habitat degradation than weak dispersers, by moving to other sites when resources become scarce.

While there is some empirical support for the prediction that weak dispersers are more vulnerable to intensification than strong dispersers (e.g. [2,12]), there are also studies suggesting the opposite—that strong dispersers are more vulnerable to intensification than weak dispersers [13–15]. If strong dispersers are more likely to leave habitat patches and enter the human-dominated portions of a landscape, they may be more susceptible to impacts such as road kill, desiccation, pesticides and hunting. For example, a meta-analysis of road impacts found that populations of more mobile birds and mammals are more susceptible to road impacts than are populations of less mobile birds and mammals [16].

Conservation decision-making often relies on a combination of local knowledge and general rules (reviewed in [17,18]). The prediction that weak dispersers are usually more vulnerable to intensification than strong dispersers has been used as one such general rule in conservation. For example, weak dispersers have been targeted as ‘focal’ species for conservation planning [19–21]. In addition, weak dispersal has been used to assess species' conservation status, based on the idea that weak dispersers are more vulnerable to stressors than strong dispersers [22].

However, to date there has been no empirical synthesis to evaluate support for this general rule. Are the cases where strong dispersers are more vulnerable than weak dispersers only rare exceptions to the general rule, or are cases where weak dispersers are most vulnerable less ubiquitous than predicted? Application of the rule in species’ risk assessments and conservation planning will result in poor conservation outcomes if dispersal ability is an unreliable predictor of species vulnerability to intensification. Our main objective is to review the empirical literature to evaluate the support for the general rule that weak dispersers are more vulnerable than strong dispersers to intensification (prediction (i)), relative to three alternatives: strong dispersers are more negatively affected by intensification than weak dispersers (prediction (ii)); intermediate dispersers are more negatively affected by intensification than weak and strong dispersers (prediction (iii)); or intermediate dispersers are less negatively affected by intensification than weak and strong dispersers (prediction (iv)). The latter two predictions take into account both the ideas that weak dispersers should be more vulnerable to habitat isolation and habitat degradation than strong dispersers, and that strong dispersers should be more vulnerable to dispersal mortality [23]. Intermediate dispersers could be most vulnerable to intensification (third prediction) if they are less able to mitigate isolation effects than strong dispersers but they also enter the matrix often enough to experience high dispersal mortality. Alternatively (fourth prediction), intermediate dispersers might avoid isolation effects better than weak dispersers and also avoid dispersal mortality better than strong dispersers.

Even if the rule that weak dispersers are more vulnerable to intensification than strong dispersers is not general, it may still be valuable to identify cases where it (or the opposite) consistently applies. As introduced above, there is theoretical and empirical support for the prediction that weaker dispersers are more vulnerable to intensification, and theoretical and empirical support for the opposite prediction. Thus, it is possible that the role of dispersal in species' responses to intensification is context specific. First, we predict that weak dispersers are more vulnerable to intensification than strong dispersers at low latitudes and the reverse at high latitudes. More dynamic environments at high latitudes should drive evolution of species that are adapted to move through disturbed and changing landscapes, and that are less susceptible to habitat isolation than species which evolved at lower latitudes [24,25]. Second, the likelihood that weak dispersers are more vulnerable to intensification should vary among taxonomic groups, because different traits may influence whether a taxon is more affected by isolation of small populations/habitat degradation than by dispersal-related mortality. Finally, the likelihood that weak dispersers are more vulnerable to intensification than strong dispersers might depend on the type of intensification. If intensification primarily increases dispersal mortality, e.g. road kill, then strong dispersers should be most vulnerable, but if intensification primarily drives habitat degradation, e.g. clearing for agriculture, then weak dispersers should be more vulnerable.

Here we review the primary literature to answer the following questions:

(i) how prevalent is the prediction that weak dispersers are more vulnerable to intensification than strong dispersers?
(ii) how often do results of empirical studies support this prediction? and
(iii) when are weak dispersers more vulnerable to land use intensification?

## 2. Methods

### (a) Literature identification and study selection

We looked for empirical studies that (i) studied effects of land use intensification on biological responses, (ii) studied responses of more than one species to intensification, and (iii) included at least one species trait identified by the author(s) as an index of dispersal ability. We identified potentially relevant primary literature via a Web of Science search (Clarivate Analytics, USA; accessed 21 April 2017; see search string in the electronic supplementary material, appendix S1). We then screened the search ‘hits’, in four steps (details in the electronic supplementary material, appendix S2). First, we reviewed the title of each record and excluded articles that were clearly off-topic. Second, we reviewed the abstract of each retained record and excluded articles that did not meet the criteria above. Third, we reviewed the introduction and methods of each retained article and again retained only the papers that fully met the three criteria above. We used this set of papers to answer question (i). Finally, we read the results of each retained paper and used the subset of studies that statistically tested for the effect of their measure(s) of dispersal ability on the relationship between a biological response and a measure of intensification, across species, to answer questions (ii) and (iii).

To maximize consistency in study selection and data extraction (see next section), a single reviewer (A.E.M.) was responsible for screening search hits and data extraction. A.E.M. reviewed and extracted data from all papers used to address questions (i–iii) twice, so that errors in initial data extraction could be identified and corrected. To further evaluate the reliability of data extracted for our review, an independent reviewer (J.K.L.) repeated data extraction for a randomly selected 60% of the papers included in our review of the findings from studies that statistically tested for the effect(s) of dispersal ability on the relationship between a biological response and a measure of intensification (questions (ii) and (iii)). See the electronic supplementary material, appendix S3 for additional details.

### (b) Data extraction and analysis

#### (i) How prevalent is the prediction that weak dispersers are more vulnerable to intensification than strong dispersers?

For each retained article after the third step (above), we extracted any hypothesis or prediction about how dispersal ability affects the relationship between a biological response and a measure of intensification. As we were interested in the authors' *a priori* predictions, we considered only hypotheses and predictions in the introduction or methods. We categorized each hypothesis/prediction into one of the following:

(i) weak dispersers are more negatively affected by intensification than strong dispersers;
(ii) strong dispersers are more negatively affected by intensification than weak dispersers;
(iii) intermediate dispersers are more negatively affected by intensification than weak and strong dispersers; or
(iv) intermediate dispersers are less negatively affected by intensification than weak and strong dispersers.

If a study included more than one of the above predictions, we recorded each prediction and included all of them in our assessment of the authors' predictions.

#### (ii) How often do results of empirical studies support the prediction that weak dispersers are more vulnerable to intensification than strong dispersers?

In the fourth step of our literature screening (above), we identified statistical tests of the interaction between dispersal ability and intensification on a biological response from the text, tables or figures in the results sections. We did not constrain our analysis to any particular set of intensification measures. Similarly, we included any type of biological response (e.g. species occurrence or abundance, species richness) that was a quantitative response to different levels of intensification. We only considered something to be a measure of dispersal ability if it was identified as such in the article.

Note that we included tests from four meta-analyses [16,26–28]. These meta-analyses investigated the effects of dispersal ability on species vulnerability to: habitat loss/fragmentation (one paper); road/traffic intensification (one); urbanization (one) and agricultural intensification and habitat/landscape homogenization (one). The tests of the effect of dispersal on species vulnerability to intensification from these meta-analyses were not redundant with tests from the other papers we found because the authors of the meta-analyses collected estimates of the effect of a measure of intensification on a biological response from previous studies, but they collated dispersal ability estimates separately, i.e. not from the studies of intensification effects. Thus, these meta-analyses constitute new analyses of the effect of dispersal ability on species' vulnerability to intensification, rather than collections of tests of the effect of dispersal on species vulnerability to intensification from previous studies. Accordingly, we used the same criteria for data extraction from meta-analyses and from primary studies. However, we note that one of the 17 papers included in the Martinson & Raupp [26] meta-analysis [29] was included separately in our review, as was one of the 75 papers [30] that was included in the Rytwinski & Fahrig [16] meta-analysis. To avoid issues of non-independence, we omitted these two papers [29,30] from subsequent analyses. We did this instead of omitting the two meta-analyses, because the meta-analyses represent stronger tests and are based on more data than the individual studies contained within them. Our conclusions were the same whether or not we included these two studies [29,30] (electronic supplementary material, figures S1–S3).

**Figure 1.**
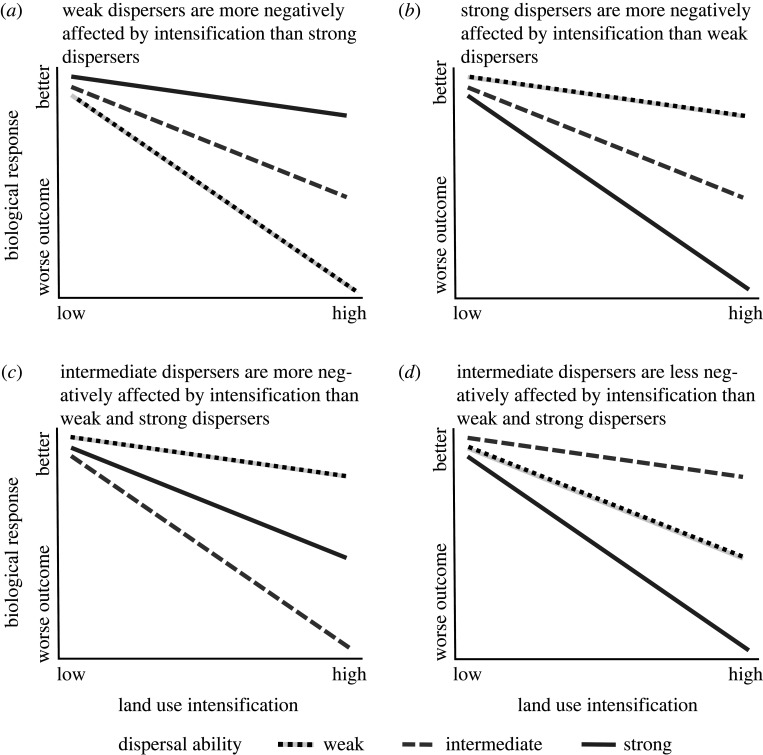
Illustration of how we identified which of four predictions was supported by a reported test of the effect of dispersal ability on the relationship between a biological response and a measure of land use intensification. Classifications were based on the relative slopes of the responses to intensification, for different levels of dispersal ability. We examined how changes in land use intensification from low intensity (e.g. low road density or high habitat amount) to high intensity (e.g. high road density or low habitat amount) were related to changes in the biological response from worse outcomes (e.g. small population or low occurrence) to better outcomes (e.g. large population or high occurrence). For example, the slopes in (*a*) are consistent with the prediction that weak dispersers are more negatively affected by intensification, because the most negative slope is for the weak dispersers.

For each test, we recorded the prediction (figure 1) with which the direction of the effect was consistent. In some cases, the authors had tested for an effect of dispersal ability on the species' response to intensification but the direction of effect was not included in the article. In these cases, if the raw data were publicly available (e.g. in electronic supplementary material or an online data repository), we replicated the analysis to get the direction of effect. Otherwise, we contacted the author(s) to request either the full results of their analyses or their raw data. Some of the reviewed articles included multiple tests of the effect of dispersal ability on the relationship between a biological response and intensification. In these cases, we used criteria detailed in the electronic supplementary material, appendix S4 to identify separate tests.

For our review, we collected only the direction of effect and not the effect size estimates needed for a meta-analysis. This decision was pragmatic and appropriate given our objectives. It was pragmatic because a meta-analysis was not possible, primarily because of the wide diversity of metrics used to index dispersal ability. We encountered at least 66 different metrics, which is understandable given the difficulty of measuring dispersal ability; however, this means that there were too few comparable effect sizes to conduct a true meta-analysis on any subset of our collected results. Thus, we used a modified ‘vote counting’ method where we tallied the tests consistent with each prediction in figure 1, irrespective of statistical significance [31]. This approach is appropriate in our situation, where the goal is to ask whether the prediction that species with weak dispersal are more vulnerable to intensification than those with strong dispersal is a valid general rule. Nevertheless, we stress that our results cannot be used to estimate an overall effect size for the relationship between dispersal ability and the influence of intensification on biological responses.

We summarized the results in two ways. First, we identified the percentage of articles consistent with each of the four predictions in figure 1. An article was considered consistent with a prediction if it contained at least one analysis consistent with that prediction. This summary at the level of articles allowed us to compare the frequency of authors' predictions (question (i)) to their findings (question (ii)). Second, we identified the percentage of tests consistent with each prediction.

#### (iii) When are weak dispersers more vulnerable to land use intensification?

For this analysis, we used the subset of findings where weak dispersers or strong dispersers were most vulnerable, i.e. figure 1*a* or *b*, because there were not enough articles consistent with predictions (iii) and (iv) to include them in this analysis (see results). For each finding, we recorded the latitude of the study, taxonomic group, type of land use intensification and type of biological response. See the electronic supplementary material, appendix S5 for details. We modelled the recorded direction of the effect from each test (1 = weak dispersers were more vulnerable to intensification, 0 = strong dispersers were more vulnerable) as a function of absolute latitude + taxonomic group + intensification type, using a generalized linear model with a binomial distribution and logit link. We included the biological response type as a fixed effect to control for effects of the response type on the probability of finding that weak dispersers are more vulnerable. Article identity was included as a random effect to account for non-independence of tests within the same study. To improve model convergence, we cubic-transformed and standardized latitude prior to analysis. For the remaining (categorical) predictors, we included only categories with more than five tests in our analysis. Our analysis was conducted in R v.3.6.3 (The R Foundation for Statistical Computing, Vienna) using the lme4 package v.1.1.23 [32]. We considered an effect strongly supported if *p* < 0.05.

## 3. Results

One hundred and forty-seven of the 1439 articles from our keyword search met the three criteria for inclusion (electronic supplementary material, figure S4). Eighty-four of these 147 articles statistically tested for an effect of some measure of dispersal ability on the relationship between a biological response and a measure of land use intensification, across species. Information needed to classify the direction of effect for all relevant statistical tests could only be obtained for 77 of this subset of 84 articles. We omitted the seven articles with missing data at this stage because we could not classify the direction of effect for the statistical test(s) within them. The retained subset of 77 papers generated a total of 425 tests of the effect of dispersal ability on species' vulnerability to intensification (median of three tests per article, range 1–54). Two studies were omitted because their findings were part of the data in meta-analyses included in this review, reducing our dataset to 75 papers and 422 tests. Five per cent (21 out of 422) of the tests came from meta-analyses. Information needed to classify the direction of effect was only directly available for 54% (227 out of 422) of the tests. We estimated the direction of effect for the remaining tests by obtaining the full results of analyses from authors or by re-analysing the raw data from the studies.

Three hundred and sixty-three tests from 73 articles were consistent with either the prediction that weak dispersers are most vulnerable to intensification or that strong dispersers are most vulnerable. For question (iii) (when are weak dispersers more vulnerable to land use intensification?), we omitted nine predictor categories with less than or equal to five tests from our analysis (see the electronic supplementary material, table S1): one taxonomic category (fish), two intensification type categories (reduction in landscape connectivity and general land use intensification) and six biological response type categories (size of geographical distribution, change in geographical distribution, a combination of population trend and change in geographical distribution, beta diversity, assessed conservation status (e.g. International Union for the Conservation of Nature red list status) and genetic diversity). Thus, our analysis for this question included 344 tests from 65 articles.

### (a) How prevalent is the prediction that weak dispersers are more vulnerable to intensification than strong dispersers?

**Figure 2.**
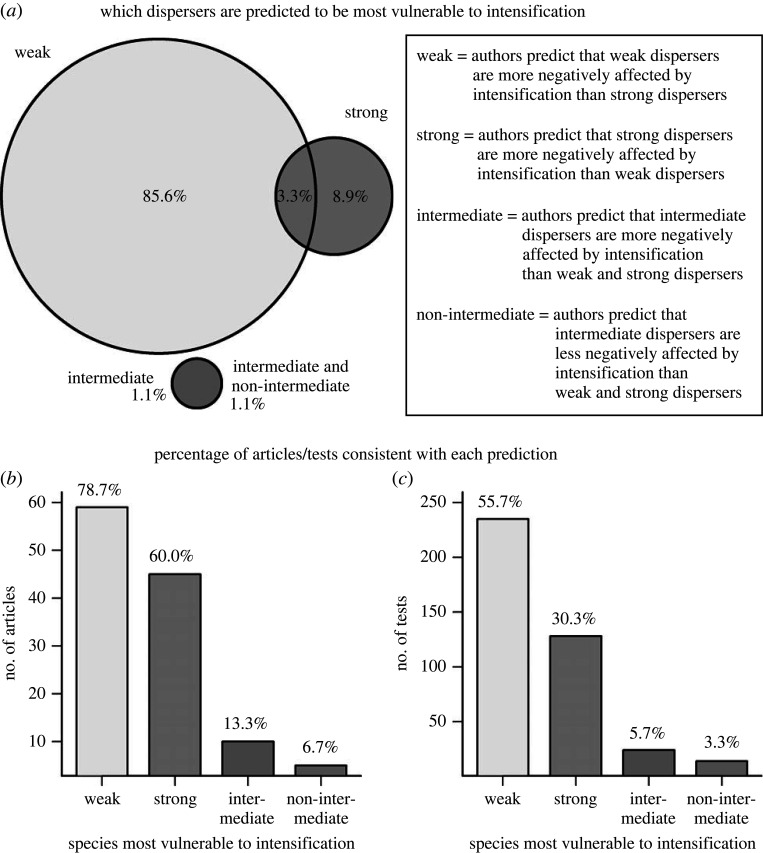
The vast majority of authors predict that weak dispersers are more vulnerable to land use intensification than strong dispersers; however, findings in support of this prediction occurred much less frequently. (*a*) The percentage of reviewed articles making each prediction: 90 out of 147 relevant articles included an explicit prediction, and four articles made multiple predictions. (*b,c*) The number of articles and tests consistent with each of the four predictions, based on 422 tests from the 75 articles with statistical tests of the effect of dispersal ability on species' responses to intensification. An article was considered consistent with a prediction if it contained at least one analysis consistent with that prediction. Values above the bars are the percentage of articles/tests consistent with each prediction. Percentages in (*b*) do not sum to 100% because some articles found support for multiple predictions. Percentages in (*c*) sum to 95%, because 5% of tests were not consistent with any of the four predictions.

Sixty-one per cent (90 out of 147) of the articles made an explicit directional prediction of the effect of dispersal ability on species responses to intensification in the introduction or methods sections. In 89% of these 90 articles, authors predicted weak dispersers to be more vulnerable to intensification than strong dispersers (figure 2*a*). Only 12% predicted the opposite—that strong dispersers would be more vulnerable to intensification—and 2% and 1% predicted that species with intermediate dispersal abilities would be most or least vulnerable, respectively (figure 2*a*). Three per cent of papers made both the prediction that weak dispersers are more vulnerable to intensification and the prediction that strong dispersers are more vulnerable. In addition, 1% of papers predicted both that species with intermediate dispersal abilities are most vulnerable to intensification and that these species are the least vulnerable.

### (b) How often do results of empirical studies support the prediction that weak dispersers are more vulnerable to intensification than strong dispersers?

Ninety-five per cent (401 out of 422) of the tests could be classified as consistent with one of the four predictions in figure 1. For the remaining tests, either the effect of dispersal ability on the response to intensification was 0 (14 results) or there was some other pattern not in figure 1 (seven results), e.g. weak dispersers were both most and least vulnerable, while strong dispersers were intermediate. Most biological response variables were species' occurrence or abundance (69%; 291 out of 422), or species richness or diversity (24%; 100 out of 422).

Seventy-nine per cent of studies reported at least one result where weak dispersers were most vulnerable to intensification, and 60% of studies found at least one result where strong dispersers were most vulnerable (figure 2*b*). Thus, while authors predicted that weak dispersers would be more vulnerable to intensification 7.3 times as often as the opposite (80 versus 11 articles, respectively), articles found that weak dispersers were more vulnerable to intensification only 1.3 times as often as the opposite (59 versus 45 articles; figure 2*b*).

Fifty-six per cent of tests found that weak dispersers were more vulnerable to intensification, and 30% of tests found that strong dispersers were more vulnerable. Thus, there were 1.8 times as many findings where weak dispersers were more vulnerable to intensification than the opposite (235 versus 128 tests, respectively; figure 2*c*).

**Figure 3.**
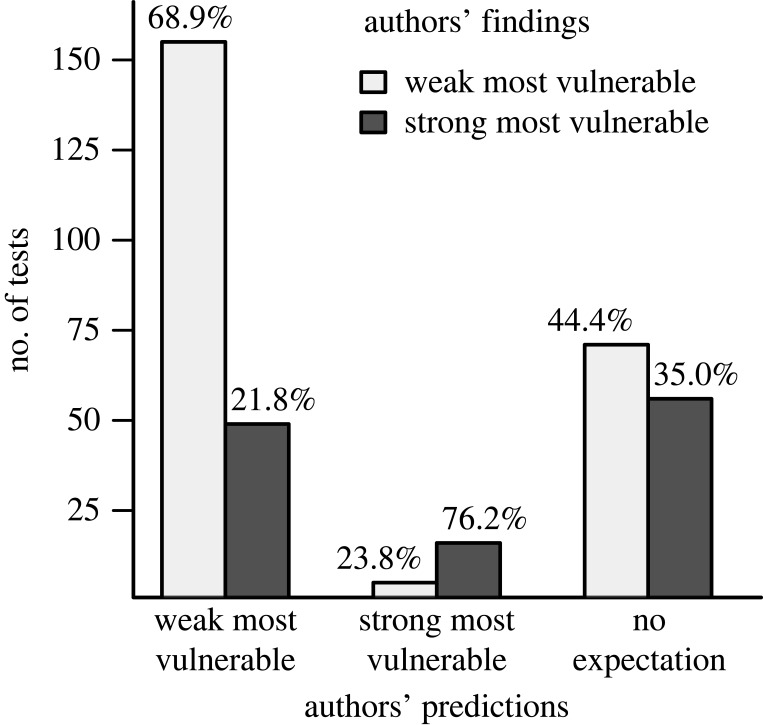
Authors who predicted that weak dispersers are more vulnerable to land use intensification than strong dispersers (44 articles) were much more likely to report findings consistent with that prediction than the reverse. Similarly, authors who predicted that strong dispersers are more vulnerable (five articles) were much more likely to report findings consistent with that prediction. There was more similar support for the two outcomes when authors did not make an *a priori* prediction (23 articles). Values above the bars are the percentage of tests in papers with a given prediction that were consistent with the prediction that weak dispersers are more vulnerable to intensification or the opposite. Percentages may not sum to 100%, because some tests were not consistent with either of these two predictions.

The 44 studies where authors predicted that weak dispersers are more vulnerable to intensification were much more likely to find this result than the reverse, and the five studies where authors predicted that strong dispersers are more vulnerable to intensification were much more likely to find this result than the reverse (figure 3). However, there were only 1.3 times as many findings where weak dispersers were more vulnerable to intensification than the opposite (71 versus 56 tests, respectively) in the 23 studies in which authors did not make an *a priori* prediction (figure 3).

### (c) When are weak dispersers more vulnerable to land use intensification?

**Figure 4.**
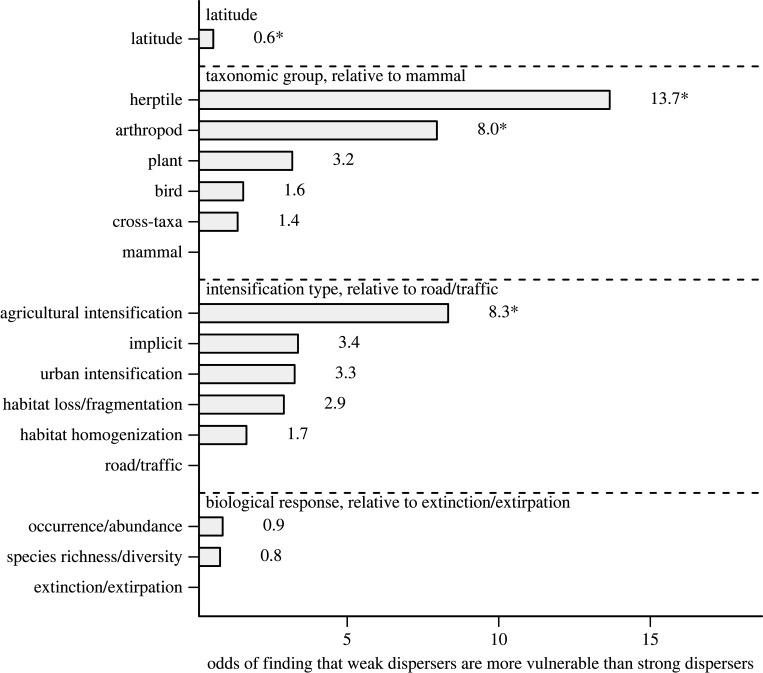
Authors were much more likely to find that weak dispersers were more vulnerable to land use intensification when studying herptiles relative to other taxa. Shown are effects of absolute latitude, taxonomic group, intensification type and the biological response type on the odds of finding that weak dispersers are more vulnerable to intensification (i.e. odds ratio estimates from the statistical model; see the electronic supplementary material, figure S5 for model coefficients). We used a generalized linear mixed effects model with a binomial distribution and logit link, including article identity as a random effect. Analysis was based on 344 tests extracted from 65 studies. *p < 0.05. Comparison of effects between all other levels of a predictor (e.g. arthropod to bird) was non-significant (see the electronic supplementary material, figure S5).

The direction of the effect from a statistical test of the effect of dispersal ability on species vulnerability to intensification depended on latitude, taxon and type of intensification (figure 4; electronic supplementary material, figure S5). Authors were more likely to find that weak dispersers were more vulnerable to intensification at low latitudes than at high latitudes, when studying herptiles and when studying agricultural intensification.

## 4. Discussion

Based on the results of this literature review, we conclude that weak dispersal ability should not be used as a general indicator of species that are vulnerable to land use intensification, or to prioritize species for conservation action. Although 79% of reviewed articles included at least one test where the direction of effect was consistent with the prediction that weak dispersers are more vulnerable to intensification than strong dispersers, 60% of reviewed articles included at least one test consistent with the opposite prediction, that strong dispersers are more vulnerable than weak dispersers. Forty-four per cent of reviewed tests were not consistent with the prediction that weak dispersers are more vulnerable to intensification.

We can only speculate as to why the prediction that weak dispersers are more vulnerable to intensification than strong dispersers is much more frequent in the literature than are findings that support it. One possibility is that the terms ‘weak dispersal’ and ‘strong dispersal’ evoke immediate assumptions of negative and positive (respectively) responses. There also may be an element of confirmation bias in the literature if authors are more likely to focus on results that support this prediction than the opposite. We found some preliminary support for this speculation by comparing the contents of abstracts to the contents of results (electronic supplementary material, appendix S5). In addition, authors who made predictions were more likely to find what they predicted than the opposite. Although this could simply be owing to authors making *post hoc* predictions to match their findings [33], it could also reflect unintentional under-reporting of results that go against predictions, for example, if authors and reviewers are more likely to question the quality of data when findings go against predictions [33]. If true, then our results from the studies with no *a priori* prediction may be more reliable than those with *a priori* predictions. Across these studies, there was more similar support for the prediction that weak dispersers were more vulnerable to intensification and the prediction that strong dispersers were more vulnerable.

We found support for the predictions that the likelihood of weak dispersers being more vulnerable to intensification: (i) is higher in lower latitudes, (ii) varies among taxa; and (iii) depends on the type of intensification. Strikingly, the odds of finding that weak dispersers were more vulnerable were 13.7 times higher for herptiles than for mammals. We speculate that differences among taxa are driven by life-history traits but further research is needed to identify those traits. Another notable finding is that the odds that weak dispersers were more vulnerable to agricultural intensification were much higher than the odds that they were more vulnerable to road intensification. We could infer that this occurs because agricultural intensification degrades habitat quality (e.g. through increased pesticide/fertilizer use), while increasing road density increases dispersal mortality. However, we acknowledge that these inferences are weak because the impacts of different types of land use intensification do not divide neatly into these categories.

We suggest two directions for future research. First, research is needed on effects of dispersal independent of other life-history attributes. Most studies measured dispersal ability using proxies that may be correlated with life-history attributes that can also influence a species' response to intensification. For example, body size is frequently used as an index of dispersal ability [34,35] but is often correlated with reproductive rate [16]; and reproductive rate has been shown to strongly influence species’ responses to habitat loss [36–38]. Thus, an effect of body size on species' responses to intensification could reflect an effect of either dispersal or reproductive rate or both. Second, more research is needed to test the predictions that intermediate dispersers are most or least negatively affected by intensification. Although we found little support for these predictions in our review, only 23% of tests in the reviewed studies (98 out of 422) were designed in a way that would allow detection of support for either of these predictions. Within this 23% of studies, 24% of tests (24 out of 98) were consistent with the prediction that intermediate dispersers are most vulnerable to intensification and 14% (14 out of 98) were consistent with the opposite.

## 5. Conclusion

Our results show that weak dispersal is not a reliable predictor of vulnerability to land use intensification, and therefore weak dispersal is not a general indicator of species risk in human-modified landscapes. We note that our conclusions are based on the direction of effect only, and on comparison of the frequency of findings consistent with the prediction that weak dispersers are more vulnerable to intensification, relative to three alternatives. While this is appropriate in our context where we are asking about the general validity of a widespread prediction, our results cannot be used to infer large (or small) effects overall or in particular situations. However, it is striking that, when authors expressed no *a priori* predictions, strong dispersers were nearly as likely as weak dispersers to be found most vulnerable to intensification. Thus, we recommend that weak dispersal should not be used as a general criterion for prioritizing species for conservation actions.

## Data Availability

Collected data and code for analyses are available on Figshare: https://doi.org/10.6084/m9.figshare.14173466 [39]. Supplementary material is available online [40].
